# NF-κB-Dependent IFIT3 Induction by HBx Promotes Hepatitis B Virus Replication

**DOI:** 10.3389/fmicb.2019.02382

**Published:** 2019-10-11

**Authors:** Fengchao Xu, Hongxiao Song, Beiying An, Qingfei Xiao, Genhong Cheng, Guangyun Tan

**Affiliations:** ^1^Department of Immunology, Institute of Translational Medicine, The First Hospital of Jilin University, Changchun, China; ^2^Department of Clinical Laboratory, The First Hospital of Jilin University, Changchun, China; ^3^Department of Nephrology, The First Hospital of Jilin University, Changchun, China; ^4^Department of Microbiology, Immunology and Molecular Genetics, University of California, Los Angeles, Los Angeles, CA, United States; ^5^Center of Systems Medicine, Institute of Basic Medical Sciences, Chinese Academy of Medical Sciences and Peking Union Medical College, Beijing, China; ^6^Suzhou Institute of Systems Medicine, Suzhou, China

**Keywords:** IFIT3, NF-κB, HBx, HBV, interferon

## Abstract

Therapeutic administration of type I IFN (IFN-I) is a common treatment option for individuals suffering from hepatitis B virus (HBV) infection. IFN-I therapy, however, has a relatively low response rate in HBV-infected patients and can induce serious side-effects, limiting its clinical efficacy. There is, thus, a clear need to understand the molecular mechanisms governing the influence of IFN-I therapy in HBV treatment in order to improve patient outcomes. In this study, we explored the interactions between HBV and IFITs (IFN-induced proteins with tetratricopeptide repeats), which are classical IFN-inducible genes. Specifically, we found that HBV patients undergoing IFN-I therapy exhibited elevated expression of IFITs in their peripheral blood mononuclear cells (PBMCs). We further observed upregulation in the expressions of IFIT1, IFIT2, and IFIT3 in cells transfected with the pHBV1.3 plasmid, which yields infectious virions in hepatic cells. We additionally found that HBx, which is the only regulatory protein encoded within the HBV genome, activates NF-κB, which in turn directly drives IFIT3 transcription. When IFIT3 was overexpressed in HepG2 cells, HBV replication was enhanced. Together, these results suggest that IFIT genes may unexpectedly enhance viral replication, thus making these genes potential therapeutic targets in patients with HBV.

## Introduction

Hepatitis B virus (HBV) infection remains a major cause of morbidity and mortality globally (Hirschman et al., 1969; Cossart and Field, 1970; Dane et al., 1970; Zanen-Lim, 1976; Glebe, 2007). Regions, where HBV is endemic, contain roughly 75% of the global population, and up to a third of these individuals have suffered HBV exposure at some point in their lives. While an HBV vaccine has been available for three decades, there are still approximately 240 million chronically HBV-infected individuals worldwide, with 39 million of these residing in Southeast Asia (Childs et al., 2018). In addition, roughly 100 million individuals in China and Africa are HBV surface antigen (HBsAg)-positive, and these individuals are at an elevated risk of suffering from liver cirrhosis or hepatocellular carcinoma (HCC) later in life (El-Serag and Rudolph, 2007; Levrero and Zucman-Rossi, 2016).

Type I interferons (IFN-I) are a group of interferon cytokines originally identified for their antiviral activity (Isaacs and Lindenmann, 1957). IFN-I mediates antiviral activity via JAK/STAT-mediated signaling through the type I IFN receptor (IFNAR1), leading to the upregulation of roughly 300 IFN-stimulated genes (ISGs) (Ivashkiv and Donlin, 2014). These ISGs mediate a range of varied antiviral activities (Liu et al., 2013; Chen et al., 2014), with many exhibiting specific efficacy against HBV infection and activity (Tan et al., 2018a). Our group and others have previously found that many ISGs are able to inhibit the replication of HBV, including APOBEC3G, TRIM25, CBFB, and TRIM14 (Xu et al., 2016, 2018; Tan et al., 2018b, c). Despite ISG induction, however, in chronically infected individuals, Hepatitis B virus is able to continuously replicate and survive within infected hepatocytes for unclear reasons. One potential explanation for this observation is that HBV-infected cells may suffer from impaired IFN-I signaling. Consistent with this hypothesis, studies have found that HBV is able to effectively infect and replicate in hepatocytes in part due to their lack of the pattern recognition receptors (PRRs) expression which are necessary to detect and respond to viral DNA (Thomsen et al., 2016). However, even when patients are directly administered IFN-I therapeutically which induces inhibitory ISGs, many patients still exhibit poor responses and outcomes, suggesting that ISG induction alone is insufficient to eliminate HBV infection. Based on these findings, we hypothesized that some among the 300 ISGs induced by IFN-I signaling, may facilitate enhanced HBV replication.

In the present study, we focused on the IFN-induced tetratricopeptide repeat (IFIT) family of classical ISGs and examined their roles in this context. IFITs have been shown to play important roles in antiviral responses (Diamond and Farzan, 2013), but their specific functions in the context of HBV replication are not fully understood. IFIT3 has been reported to be an effective marker of patient outcomes in those undergoing interferon therapy, with high hepatic IFIT3 expression predicting better outcomes (Yang et al., 2017). IFIT3 was further reported to stabilize IFIT1, modulating the RNA-binding domains of IFIT1 and giving rise to the specific recognition of the viral RNA (Johnson et al., 2018). In the present study, we found that when HepG2 cells were transfected with the pHBV1.3 plasmid, they exhibited increased expression of IFIT1, IFIT2, and IFIT3, whereas IFIT5 expression was reduced. Interestingly, we found that IFIT3 enhanced HBV replication following pHBV1.3 transfection, and in these infected cells, we found that HBx promoted the activation of NF-κB, which in turn induced IFIT3 expression. Together, these findings suggest that IFIT3 promotes rather than suppress HBV replication in hepatocytes, making it a potential therapeutic target for the treatment of HBV infection.

## Materials and Methods

### Cell Culture, Plasmids, and Reagents

HEK293T and HepG2 cells were grown in DMEM supplemented with 10% heat-inactivated fetal bovine serum (FBS), penicillin (100 IU/mL), and streptomycin (100 mg/mL) in a 5% CO_2_ incubator at 37°C. siRNA targeting p65 was purchased from Cell signaling technology (SignalSilence NF-κB p65 siRNA II #6534). An IFIT3 expression construct was generated via cloning the coding region sequence into the VR1012 expression vector. Antibodies specific for the Flag-tag were obtained from Sigma (F4049, United States). Antibodies specific for GAPDH were obtained from Proteintech (10494-1-AP). Antibodies specific for IFIT2 (#39584), IFIT3 (#22040), and IFIT5 (#35225-2) were purchased from Signalway. IFIT1 and p65 were purchased from CST (#14769, # 8242). An antibody specific for HBc was purchased from Abcam (ab8638).

### Quantitative Real-Time PCR

An EasyPure RNA Kit (ER01-01,Transgen, China) was used to extract total cellular RNA based on manufacturer’s instructions, after which cDNA was generated using a TransScript First-Strand cDNA Synthesis SuperMix Kit (AT341-02, Transgen, China). Supernatant HBV DNA was obtained based on kit instructions provided with the EasyPure Viral DNA/RNA Kit (ER201-01, Transgen, China). For all qPCR experiments, GAPDH served as a normalization control, and we quantified gene expression as in previous studies (Tan et al., 2012). Primer sequences used in this study are compiled in Supplementary Table S1.

### Western Blotting

We performed western immunoblotting as in previous studies (Tan et al., 2017). Briefly, ice-cold cell lysis buffer (20 mM HEPES, 350 mM NaCl, 20% glycerol, 1% NP40, 1 mM MgCl2, 0.5 mM EDTA, 0.1 mM EGTA, 0.5 mM DTT) was used to lyse cells for 30 min with agitation. After extraction, protein levels were quantified with the Coomassie Plus^TM^ protein assay reagent (Thermo Fisher Scientific). Proteins were then loaded and separated via SDS-PAGE, followed by transfer onto PVDF membranes. Blots were blocked using 5% skim milk in TBST, after which appropriate antibodies were used for protein detection. A ChemiDoc^TM^ XRS^+^ Molecular Imager software (Bio-Rad) was used to quantify protein band densities.

### Enzyme-Linked Immunosorbent Assay

At 72 h post-transfection with pHBV1.3 and IFIT3 expression plasmids or mock transfection, cell supernatants were collected and HBeAg and HBsAg levels were assessed via ELISA according to the instructions provided with a commercial kit (Kehua Shengwu, China).

### CRISPR/Cas9 Knockout

HepG2 cells were plated in 24-well plates for 16 h, after which they were transfected with plasmids encoding a puromycin-resistance gene as well as Cas9 and an IFIT3-targeting sgRNA using the Viafect transfection reagent (E4982, Promega). Puromycin selection (2 ug/mL) was initiated 36 h after transfection, after which cells were assessed with IFIT3-specific antibodies via western blotting. After a 2-day selection period, single-cell cloning was performed via serial dilution, with clonal cells plated and cultured in 96-well plates. After clonal lines had proliferated sufficiently, IFIT3 knockout was again confirmed via western blotting, and this result was further confirmed through DNA sequencing of selected clones. The sgRNA sequences used are given in Supplementary Table S1.

### IFIT3 Promoter Reporters and Dual-Luciferase Reporter Assay

In order to construct IFIT3 promoter reporters, the IFIT3 promoter region was cloned into the pGL4.11 expression vector, with mutated promoter sequences being generated using the Quick-Change PCR primers (Supplementary Table S1). These wild-type (WT) or mutant IFIT3 promoters were then transfected into HEK293T cells along with the pGL4.74 Tk-Rluc reporter, with or without plasmids encoding HBx. After 24 h, cells were lysed using a passive lysis buffer, and a Dual-Luciferase Reporter Assay System (E1910, Promega) was used to measure firefly and Renilla luciferase activity in these samples.

### Chromatin Immunoprecipitation

Chromatin immunoprecipitation (ChIP) assays were carried out as described previously (Sato et al., 2015). Briefly, the treated cells were cross-linked with 1% formaldehyde, sheared to an average size of ∼500 bp, and immunoprecipitated with immunoglobulin G (IgG) or antibodies against p65. The ChIP-PCR primers (Supplementary Table S1) were designed to amplify the proximal promoter regions containing putative p65-binding sites within the IFIT3 promoter as illustrated (Figure 3).

### Samples

We enrolled a total of eight HCC patients with HBV infection in the present study who were undergoing IFN-α treatment for the first time (Supplementary Table S2). Venous blood samples were obtained from these patients 15 min prior to IFN-α administration, as well as 24, 48, 96, 168, and 240 h following treatment. These blood samples were then used to isolate PBMCs by Ficoll density gradient separation for processing. The IRB of Jilin University, the First Hospital approved this study.

### Statistical Analysis

Data are presented as means ± SD, and the observations were compared by Students *t*-tests. *P* < 0.05 was the threshold for statistical significance.

## Results

### IFNα-Treated HBV Patients Exhibit IFIT Expression

**FIGURE 1:**
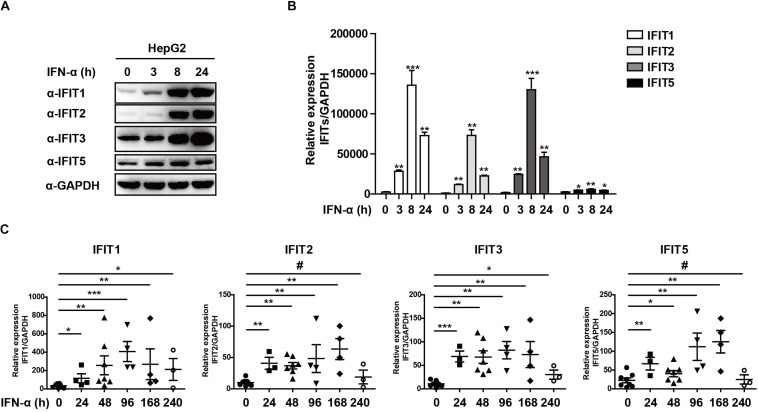
IFNα-treated HBV patients exhibit IFIT expression. **(A,B)** HepG2 cells were plated in 12-well plates and treated using 10 ng/mL IFNa. The expression of IFIT1, IFIT2, IFIT3, IFIT5, and GAPDH was then assessed via western blotting and qPCR. **(C)** PBMCs were collected from patients with HBV that were undergoing IFNα therapy (control:8, 24 h:4, 48 h:6, 96 h:4, 168 h:4, 240 h:3). Expression of IFIT1, IFIT2, IFIT3, and IFIT5 in these cells was assessed via qPCR. Data are means ± SD of triplicate experiments, and were compared via Student’s *t*-test. ^#^*p* > 0.5, ^∗^*P* < 0.05, ^∗∗^*P* < 0.01, ^∗∗∗^*p* < 0.001.

As classical ISGs, IFITs have been found to play key antiviral roles against a number of viral pathogens, leading us to assess their relevance in the context of HBV infection (Pichlmair et al., 2011; Diamond and Farzan, 2013; Katibah et al., 2013; Johnson et al., 2018). Following HepG2 cells stimulation with IFNα, we found that IFIT1, IFIT2, IFIT3, and IFIT5 mRNA and protein expressions were increased significantly (Figures 1A,B). We next assessed the expression pattern of these same genes in patients suffering from HBV infections by collecting PBMCs from both controls and HBV patients undergoing IFNα therapy. Consistent with our *in vitro* findings, we observed a comparable upregulation of these IFIT proteins in response to IFN stimulation in patient samples compared to controls (Figure 1C). Together, these results indicate that IFNα therapy in HBV patients leads to the upregulation of IFIT proteins.

### HBx Regulates IFIT Protein Expression

**FIGURE 2:**
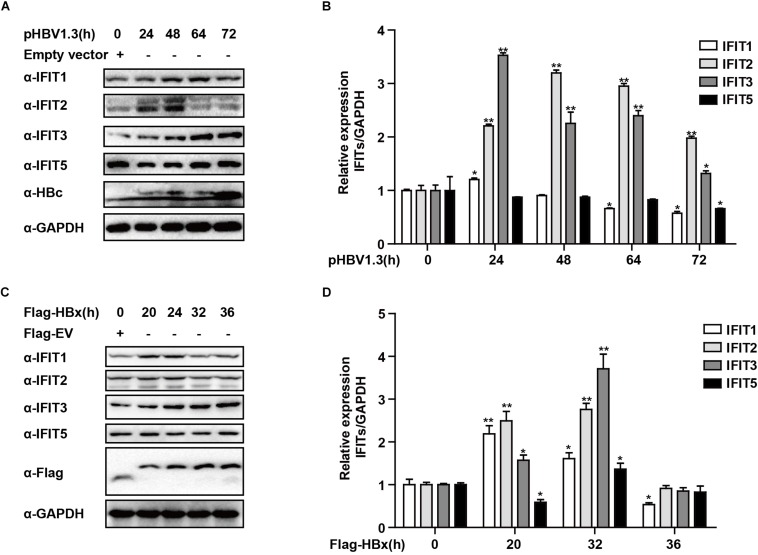
HBx is a key regulator of IFIT expression. **(A,B)** pHBV1.3 or Empty vector was transfected into HepG2 cells at the indicated time points, after which cells were analyzed by qPCR and Western blotting to assess the expression of IFIT1, IFIT2, IFIT3, IFIT5, and GAPDH. **(C,D)** After transfection with Flag-HBx or Flag-EV (empty vector) at the indicated time points, cells were analyzed by qPCR and Western blotting to assess the expression of IFIT1, IFIT2, IFIT3, IFIT5, and GAPDH. Data are means ± SD of triplicate experiments, and were compared via Student’s *t*-test. ^∗^*P* < 0.05, ^∗∗^*P* < 0.01.

While IFITs being induced upon IFN treatment was not unexpected, their specific role in liver cells in the context of HBV replication remains poorly understood. We, therefore, transfected HepG2 cells with the pHBV1.3 plasmid in order to generate infectious HBV and found that this led to the upregulation of IFIT1, IFIT2, and IFIT3 in these cells. IFIT1 and IFIT2 were both induced at early time points and decreased at later time points, while although IFIT3 was rapidly induced at the mRNA level, the IFIT3 protein levels did not decline till the 72 h time point. IFIT5 was not upregulated at either the mRNA or protein level (Figures 2A,B). As we and others have previously reported, HBV infection can induce the production of type III IFNs, whereas it fails to induce type I or II IFN production (Sato et al., 2015; Xu et al., 2018). We, therefore, assessed whether type III IFNs were responsible for the upregulation of IFITs in HBV-replicated cells. Unlike CBFβ, even after neutralizing these type III IFNs, we found that IFIT3 was still induced in HepG2 cells following pHBV1.3 transfection (Supplementary Figure S1), suggesting that these proteins are likely regulated by other factors within the host cells, or by HBV-associated proteins. The viral factor HBx is known to be expressed in patients suffering from both acute and chronic hepatitis, and it is also thought to play key roles in regulating HBV replication and modulating the expression of genes in infected host cells (Zhang et al., 2004; Kremsdorf et al., 2006; Slagle and Bouchard, 2018). As such, we next explored whether HBx was able to modulate IFIT expression, by overexpressing HBx in HepG2 cells. Through this approach, we found that IFITs were significantly induced at the mRNA and protein level (Figures 2C,D), suggesting that HBx plays a key role in inducing IFIT expression upon HBV infection.

### HBx-Mediated NF-κB Activation Drives IFIT3 Induction

**FIGURE 3:**
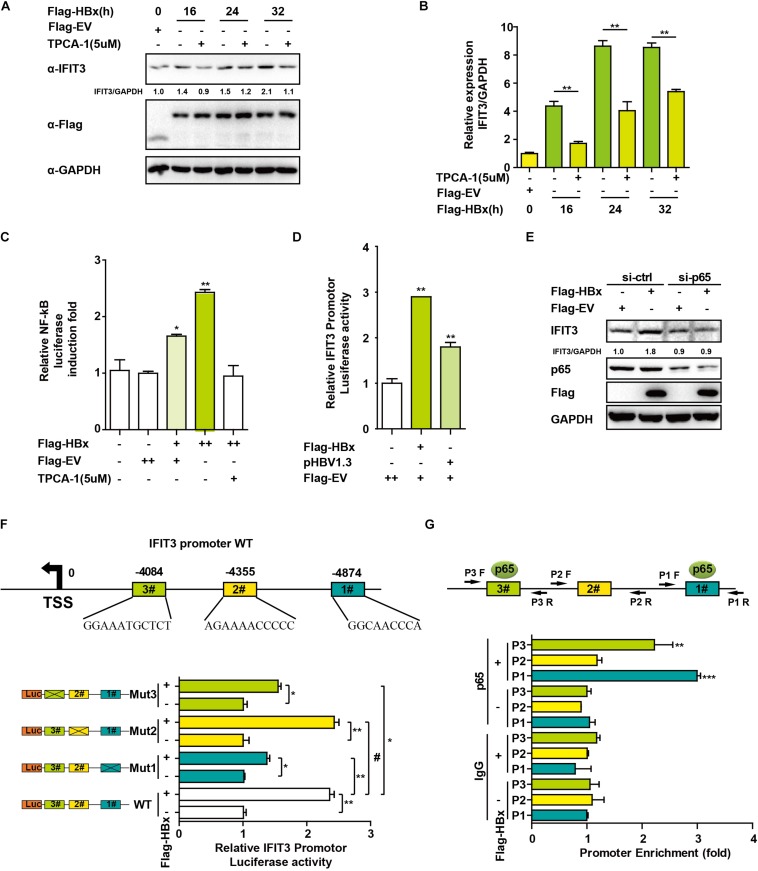
HBx induces IFIT3 expression in an NF-κB dependent fashion. **(A,B)** After transfection with Flag-HBx or Flag-EV (empty vector) at the indicated time points, HepG2 cells were treated with TPCA-1 (5 um/mL) as indicated. Cells were then analyzed by qPCR or Western blotting to assess IFIT3, Flag, or GAPDH. **(C)** HepG2 cells were transfected with Flag-HBx and NF-κB luciferase reporter as indicated, 16 h later, cells were treated with TPCA-1 for 3 h, and cells were collected and NF-κB luciferase activity was measured. **(D)** 24 h after transfection with Flag-HBx, pHBV1.3 or an empty vector, HepG2 cells were used to assess IFTI3 promoter luciferase activity. **(E)** HepG2 cells were transfected with p65 siRNA or control siRNA, and then 24 h later Flag-HBx was transfected as indicated, and cells were collected after another 36 h and subjected to immunoblotting with p65, IFIT3, Flag or GAPDH antibodies. **(F)** Diagram indicating putative NF-κB binding sites in the IFIT3 promoter region. Luciferase reporters encoding WT or mutated IFIT3 promoter regions were transfected into HepG2 cells along with Flag-HBx and the pGL4.7 TK-Luc reporter, and IFTI3 promoter luciferase activity was assessed 24 h following transfection. **(G)** HepG2 cells were transfected with Flag-HBx or EV as indicated, and 48 h later a ChIP assay was conducted to analyze p65 binding to sites (#1 and #3) within the IFIT3 promoter region. IgG was used as a negative control. Data are means ± SD of triplicate experiments, and were compared via Student’s *t*-test. ^∗^*P* < 0.05, ^∗∗^*P* < 0.01, ^∗∗∗^*p* < 0.001.

We next sought to assess how HBx induces and regulates IFIT3 expression, given that in HBV-infected patients, IFIT3 serves as an important marker of IFN therapy (Yang et al., 2017). HBx is known to activate NF-κB signaling (Waris et al., 2001), and consistent with this we found that treating the cells with TPCA-1, which inhibits NF-κB activation, led to a reduction in IFIT3 induction in response to HBx overexpression (Figures 3A,B). We further confirmed that HBx-mediated NF-κB activation using a luciferase reporter system, and the HBx-induced NF-κB activation was inhibited by TPCA-1 (Figure 3C), and found that HBx or pHBV1.3 plasmid transfection led to a significant increase in IFIT3 promoter activity (Figure 3D). We further confirmed these results by knocking down p65 with a specific siRNA. As expected, HBx-induced IFIT3 expression was inhibited in the p65 knockdown cells (Figure 3E and Supplementary Figure S2A). These findings were consistent with a model wherein HBx induces IFIT3 promoter activation in an NF-κB-dependent manner. To confirm this possibility, we mutated the putative NF-κB binding sites in the IFIT3 promoter region [predicted using JASPAR (Mathelier et al., 2016)], and found that mutations at both sites #1 and #3 led to a significant disruption in NF-κB binding to the IFIT3 promoter, whereas site #2 mutations had no effect (Figure 3E). In addition, a ChIP assay confirmed that p65 was able to bind to site #1 and #3 (Figure 3G). Together, these findings indicate a key role for NF-κB in the HBx-mediated expression of IFIT3.

### IFIT3 Promotes HBV Replication

**FIGURE 4:**
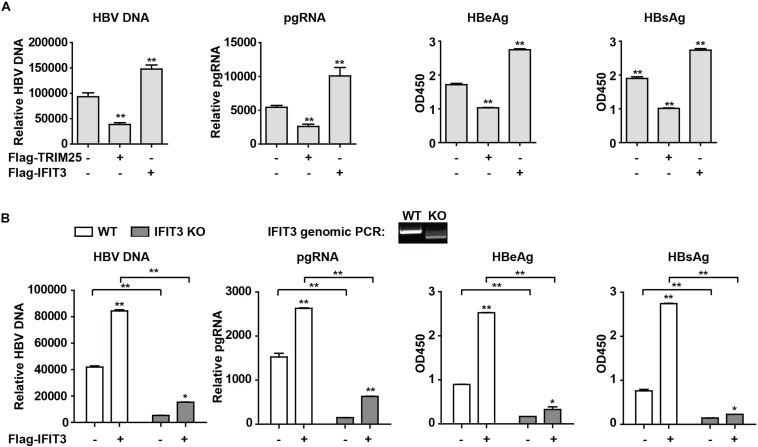
IFIT3 promotes HBV replication. **(A,B)** HepG2 cells were transfected with pHBV1.3, Flag-Trim25, Flag-IFIT3, or an empty vector, and after 72 h cells supernatants were collected. qPCR was used to measure cellular HBV DNA and pgRNA, while supernatant HBeAg and HBsAg levels were measured via ELISA. Data are means ± SD of triplicate experiments, and were compared via Student’s *t*-test. ^∗^*P* < 0.05, ^∗∗^*P* < 0.01. **(C)** WT or IFIT3-KO HepG2 cells were transfected with pHBV1.3, or were co-transfected with pHBV1.3 and IFIT3 as indicated. At 72 h post-transfection, qPCR was used to measure cellular HBV DNA and pgRNA, while supernatant HBeAg and HBsAg levels were measured via ELISA. Data are means ± SD of triplicate experiments, and were compared via Student’s *t*-test. ^∗^*P* < 0.05, ^∗∗^*P* < 0.01.

Given that IFIT3 can be induced both by host factors (IFN) and viral factors (HBx), we next sought to explore its functional role in the context of HBV viral replication. Given that we have previously found TRIM25 to be able to restrict the replication of HBV, we used this factor as a positive control and transfected cells with pHBV1.3 along with either IFIT3 or TRIM25. Interestingly, we found that TRIM25 and IFIT3 played opposing roles in modulating HBV replication (Figure 4A and Supplementary Figure S2B). And we found that IFIT2 slightly enhanced HBV replication, while both IFIT1 and IFIT5 inhibited HBV replication (Supplementary Figures S2C,D). In addition, we used CRISPR/Cas9 technology to knock out IFIT3 expression in HepG2 cells (Supplementary Figure S2E, line 1 and 3) and then confirmed that HBV replication in these cells was markedly reduced as compared to WT HepG2 cells, whereas transfecting IFIT3 back into these IFIT3-KO cells rescued HBV replication (Figure 4B and Supplementary Figure S2E). Together, these findings clearly reveal that IFIT3 serves to promote HBV replication, in contrast to the traditional roles played by most known ISGs.

## Discussion

Hepatitis B virus remains a major threat to human health, yet treatment with IFN-I is largely ineffective and patients suffer from high rates of substantial side effects. In the present study, we sought to explore the expression and function of the classical ISG IFIT3 in the context of HBV infections, while unexpectedly revealing it to promote viral replication. IFN treatment in HBV patients led to robust IFIT upregulation, while viral infection of liver cells also led to direct IFN-independent IFIT3 upregulation. This IFIT3 expression was at least in part induced via HBx-mediated activation of the transcription factor NF-κB, which subsequently bound to the IFIT3 promoter and drove its transcription. These results together highlight opposing roles for certain ISGs in the context of HBV viral infection, suggesting that IFIT3 may represent a novel target for the treatment of HBV infections in addition to providing additional insight into the mechanistic reasons for the failure of IFN-I therapy in many HBV patients.

IFN signaling induces the expression of roughly 300 different ISGs, which primarily exhibit potent antiviral effector activities (Liu et al., 2012). IFITs are known to be strongly induced upon viral infection (Wacher et al., 2007; Diamond and Farzan, 2013), and they have been found to interact with the eIF3 proteins to limit viral mRNA translation for a range of virus types (Terenzi et al., 2006). Furthermore, IFIT1 has been proposed to directly recognize 5′-triphosphate viral RNA, as host mRNAs lack this feature (Pichlmair et al., 2011). IFIT1, IFIT2, IFIT3, and IFIT5 (ISG56, ISG54, ISG60, and ISG58, respectively) are the four members of the IFIT family that have been characterized. IFIT1 was the first of these proteins found to be capable of identifying viral mRNA (Pichlmair et al., 2011), and further work has suggested that it can bind to IFIT3 in order to increase its stability and specificity (Johnson et al., 2018). IFIT5 has been found to be able to bind to certain endogenous 5′-PPP-RNAs, such as tRNAs (Katibah et al., 2013). While few reports have focused on IFIT expression in HBV infected patients, IFIT3 expression has been found to be a reliable marker of patient outcomes in those undergoing interferon therapy. Interestingly, in this study higher IFIT3 expression was associated with better patient outcomes (Yang et al., 2017), whereas we found that IFIT3 overexpression enhanced HBV replication. Furthermore, we found that HBx, which is essential for normal HBV replication (Hodgson et al., 2012), and which activates NF-κB (Waris et al., 2001), drove direct NF-κB binding to the IFIT3 promoter to induce IFIT3 expression. Our work suggests that there is crosstalk between the HBV and IFN signaling pathways, with IFN-mediated and IFN-independent IFIT3 induction enhancing, rather than impairing, HBV replication. Further research will be needed to fully understand the mechanisms whereby such HBV/IFIT3 crosstalk function in enhancing viral replication. One study has found that IFIT3 can bind to signal transducer and activator of transcription 1 (STAT1) and STAT2, thereby enhancing STAT1/STAT2 hetero-dimerization and nuclear translocation in those being treated with IFNα (Yang et al., 2017). When IFIT3 is overexpressed but basal STAT1 activation remains low, it is possible that IFIT3 primarily serves to regulate other signaling pathways, thus explaining the seemingly contradictory results in these two studies. We also found that all IFITs with the exception of IFIT5 were induced upon HBV replication HepG2 cells, and as such these additional IFITs warrant further study.

**FIGURE 5:**
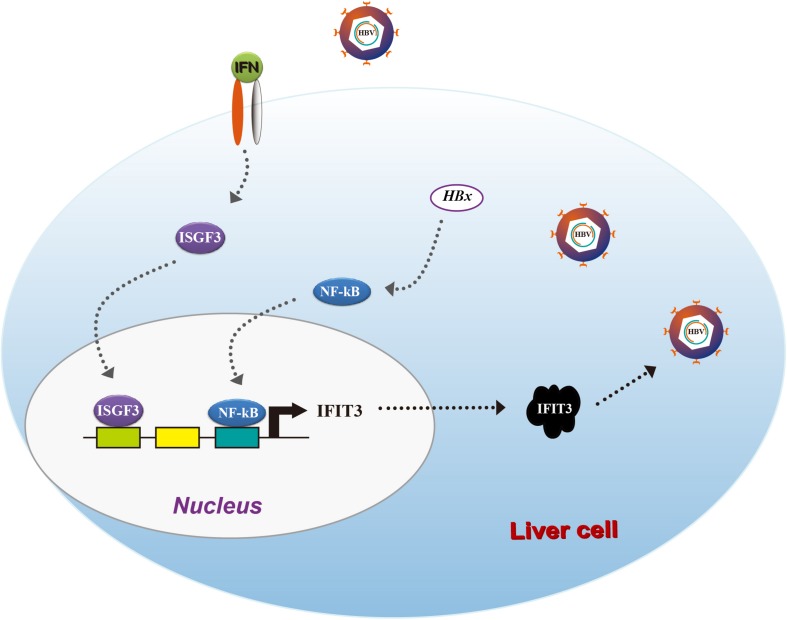
A model of the IFIT3-mediated crosstalk between IFN-I and HBV.

Taken together, our results provide clear evidence for IFIT3 as a factor, which is induced by HBx in an NF-κB-dependent manner, leading to enhanced HBV replication (Figure 5). Our results suggest that HBV patients resistant to IFN therapy may benefit from therapeutic efforts targeting IFIT3 expression.

## Data Availability Statement

All datasets generated for this study are included in the manuscript/Supplementary Files.

## Ethics Statement

The studies involving human participants were reviewed and approved by the IRB of Jilin University, the First Hospital. Written informed consent for participation was not required for this study in accordance with the national legislation and the institutional requirements.

## Author Contributions

FX performed the experiments and wrote the manuscript. HS performed the experiments and revised the manuscript. BA and QX collected the patient samples. GC gave suggestions. GT planned, designed, and performed the experiments, and wrote the manuscript.

## Conflict of Interest

The authors declare that the research was conducted in the absence of any commercial or financial relationships that could be construed as a potential conflict of interest.

## Supplementary Material

FIGURE S1 **(A,B)** pHBV1.3 plasmids were transfected into HepG2 cells, and after 42 h these cells were administered with an IFN-γ neutralizing antibody for 6 h, after which qPCR and western blotting were performed to assess the expression of the indicated genes of interest. Data are means ± SD of triplicate experiments, and were compared via Student’s *t*-test. ^∗^*P* < 0.05, ^∗∗^*P* < 0.01.

FIGURE S2 **(A)** IFIT3 mRNA expression was analyzed by Q-PCR. Data are means ± SD of triplicate experiments, and were compared via Student’s *t*-test. ^∗^*P* < 0.05, ^∗∗^*P* < 0.01. **(B)** HepG2 cells were transfected with pHBV1.3 or together with Flag-IFIT1, IFIT2, or IFIT5, and after 72 h cells supernatants were collected. qPCR was used to measure cellular HBV DNA and pgRNA, while supernatant HBeAg levels were measured via ELISA. Data are means ± SD of triplicate experiments, and were compared via Student’s *t*-test. ^∗^*P* < 0.05, ^∗∗^*P* < 0.01. **(C)** Samples from **B** were immunoblotted with Flag or GAPDH antibodies. **(D)** Samples from Figure 4A were immunoblotted with Flag or GAPDH antibodies. **(E)** Samples from Figure 4B were immunoblotted with IFIT3 or GAPDH antibodies.

TABLE S1 qPCR Primers.

TABLE S2 Hepatitis B virus infected patients with IFN treatment.

## References

[B1] Chen Y. Wang S. Yi Z. Tian H. Aliyari R. Li Y. (2014). Interferon-inducible cholesterol-25-hydroxylase inhibits hepatitis C virus replication via distinct mechanisms. *Sci. Rep.* 4:7242. 10.1038/srep07242 25467815 PMC4252895

[B2] Childs L. Roesel S. Tohme R. A. (2018). Status and progress of hepatitis B control through vaccination in the South-East Asia region, 1992-2015. *Vaccine* 36 6–14. 10.1016/j.vaccine.2017.11.027 29174317 PMC5774012

[B3] Cossart Y. E. Field A. M. (1970). Virus-like particles in serum of patients with Australia-antigen-associated hepatitis. *Lancet* 1:848. 10.1016/s0140-6736(70)92460-8 4191481

[B4] Dane D. S. Cameron C. H. Briggs M. (1970). Virus-like particles in serum of patients with Australia-antigen-associated hepatitis. *Lancet* 1 695–698. 4190997 10.1016/s0140-6736(70)90926-8

[B5] Diamond M. S. Farzan M. (2013). The broad-spectrum antiviral functions of IFIT and IFITM proteins. *Nat. Rev. Immunol.* 13 46–57. 10.1038/nri3344 23237964 PMC3773942

[B6] El-Serag H. B. Rudolph K. L. (2007). Hepatocellular carcinoma: epidemiology and molecular carcinogenesis. *Gastroenterology* 132 2557–2576. 17570226 10.1053/j.gastro.2007.04.061

[B7] Glebe D. (2007). Recent advances in hepatitis B virus research: a German point of view. *World J. Gastroenterol.* 13 8–13. 17206750 10.3748/wjg.v13.i1.8 PMC4065879

[B8] Hirschman R. J. Shulman N. R. Barker L. F. Smith K. O. (1969). Virus-like particles in sera of patients with infectious and serum hepatitis. *JAMA* 208 1667–1670. 4305624

[B9] Hodgson A. J. Hyser J. M. Keasler V. V. Cang Y. Slagle B. L. (2012). Hepatitis B virus regulatory HBx protein binding to DDB1 is required but is not sufficient for maximal HBV replication. *Virology* 426 73–82. 10.1016/j.virol.2012.01.021 22342275 PMC3294142

[B10] Isaacs A. Lindenmann J. (1957). Virus interference. I. the interferon. *Proc. R. Soc. Lond. B Biol. Sci.* 147 258–267. 26297790

[B11] Ivashkiv L. B. Donlin L. T. (2014). Regulation of type I interferon responses. *Nat. Rev. Immunol.* 14 36–49. 24362405 10.1038/nri3581 PMC4084561

[B12] Johnson B. VanBlargan L. A. Xu W. White J. P. Shan C. Shi P. Y. (2018). Human IFIT3 modulates IFIT1 RNA binding specificity and protein stability. *Immunity* 48 487–499. 10.1016/j.immuni.2018.01.014 29525521 PMC6251713

[B13] Katibah G. E. Lee H. J. Huizar J. P. Vogan J. M. Alber T. Collins K. (2013). tRNA binding, structure, and localization of the human interferon-induced protein IFIT5. *Mol. Cell.* 49 743–750. 10.1016/j.molcel.2012.12.015 23317505 PMC3615435

[B14] Kremsdorf D. Soussan P. Paterlini-Brechot P. Brechot C. (2006). Hepatitis B virus-related hepatocellular carcinoma: paradigms for viral-related human carcinogenesis. *Oncogene* 25 3823–3833. 16799624 10.1038/sj.onc.1209559

[B15] Levrero M. Zucman-Rossi J. (2016). Mechanisms of HBV-induced hepatocellular carcinoma. *J. Hepatol.* 64 (1 Suppl), S84–S101. 27084040 10.1016/j.jhep.2016.02.021

[B16] Liu S. Y. Aliyari R. Chikere K. Li G. Marsden M. D. Smith J. K. (2013). Interferon-inducible cholesterol-25-hydroxylase broadly inhibits viral entry by production of 25-hydroxycholesterol. *Immunity* 38 92–105. 10.1016/j.immuni.2012.11.005 23273844 PMC3698975

[B17] Liu S. Y. Sanchez D. J. Aliyari R. Lu S. Cheng G. (2012). Systematic identification of type I and type II interferon-induced antiviral factors. *Proc. Natl. Acad. Sci. U.S.A.* 109 4239–4244. 10.1073/pnas.1114981109 22371602 PMC3306696

[B18] Mathelier A. Fornes O. Arenillas D. J. Chen C. Y. Denay G. Lee J. (2016). JASPAR 2016: a major expansion and update of the open-access database of transcription factor binding profiles. *Nucleic Acids Res.* 44 D110–D115. 10.1093/nar/gkv1176 26531826 PMC4702842

[B19] Pichlmair A. Lee H. J. Huizar J. P. Vogan J. M. Alber T. Collins K. (2011). IFIT1 is an antiviral protein that recognizes 5’-triphosphate RNA. *Nat. Immunol.* 12 624–630. 10.1038/ni.2048 21642987

[B20] Sato S. Li K. Kameyama T. Hayashi T. Ishida Y. Murakami S. (2015). The RNA sensor RIG-I dually functions as an innate sensor and direct antiviral factor for hepatitis B virus. *Immunity* 42 123–132. 10.1016/j.immuni.2014.12.016 25557055

[B21] Slagle B. L. Bouchard M. J. (2018). Role of HBx in hepatitis B virus persistence and its therapeutic implications. *Curr. Opin. Virol.* 30 32–38. 10.1016/j.coviro.2018.01.007 29454995 PMC5988931

[B22] Tan G. Niu J. Shi Y. Ouyang H. Wu Z. H. (2012). NF-kappaB-dependent microRNA-125b up-regulation promotes cell survival by targeting p38alpha upon ultraviolet radiation. *J. Biol. Chem.* 287 33036–33047. 22854965 10.1074/jbc.M112.383273 PMC3463348

[B23] Tan G. Song H. Xu F. Cheng G. (2018a). When hepatitis B virus meets interferons. *Front. Microbiol.* 9:1611. 10.3389/fmicb.2018.01611 30072974 PMC6058040

[B24] Tan G. Xiao Q. Song H. Ma F. Xu F. Peng D. (2018b). Type I IFN augments IL-27-dependent TRIM25 expression to inhibit HBV replication. *Cell. Mol. Immunol.* 15 272–281. 10.1038/cmi.2016.67 28194021 PMC5843613

[B25] Tan G. Xu F. Song H. Yuan Y. Xiao Q. Ma F. (2018c). Identification of TRIM14 as a type I IFN-stimulated gene controlling hepatitis B virus replication by targeting HBx. *Front. Immunol.* 9:1872. 10.3389/fimmu.2018.01872 30150992 PMC6100580

[B26] Tan G. Xiao Q. Song H. Ma F. Xu F. Peng D. (2017). Type I IFN augments IL-27-dependent TRIM25 expression to inhibit HBV replication. *Cell Mol. Immunol.* 287 33036–33047. 10.1038/cmi.2016.67 28194021 PMC5843613

[B27] Terenzi F. Hui D. J. Merrick W. C. Sen G. C. (2006). Distinct induction patterns and functions of two closely related interferon-inducible human genes. ISG54 and ISG56. *J. Biol. Chem.* 281 34064–34071. 16973618 10.1074/jbc.M605771200

[B28] Thomsen M. K. Nandakumar R. Stadler D. Malo A. Valls R. M. Wang F. (2016). Lack of immunological DNA sensing in hepatocytes facilitates hepatitis B virus infection. *Hepatology.* 64 746–759. 10.1002/hep.28685 27312012

[B29] Wacher C. Müller M. Hofer M. J. Getts D. R. Zabaras R. Ousman S. S. (2007). Coordinated regulation and widespread cellular expression of interferon-stimulated genes (ISG) ISG-49, ISG-54, and ISG-56 in the central nervous system after infection with distinct viruses. *J. Virol.* 81 860–871. 17079283 10.1128/JVI.01167-06 PMC1797448

[B30] Waris G. Huh K. W. Siddiqui A. (2001). Mitochondrially associated hepatitis B virus X protein constitutively activates transcription factors STAT-3 and NF-kappa B via oxidative stress. *Mol. Cell. Biol.* 21 7721–7730. 11604508 10.1128/MCB.21.22.7721-7730.2001 PMC99943

[B31] Xu F. Song H. Li N. Tan G. (2016). HBsAg blocks TYPE I IFN induced up-regulation of A3G through inhibition of STAT3. *Biochem. Biophys. Res. Commun.* 473 219–223. 10.1016/j.bbrc.2016.03.082 27003258

[B32] Xu F. Song H. Xiao Q. Li N. Zhang H. Cheng G. (2018). Type III interferon-induced CBFbeta inhibits HBV replication by hijacking HBx. *Cell. Mol. Immunol.* 16 357–366. 10.1038/s41423-018-0006-2 29523836 PMC6461963

[B33] Yang Y. Zhou Y. Hou J. Bai C. Li Z. Fan J. (2017). Hepatic IFIT3 predicts interferon-alpha therapeutic response in patients of hepatocellular carcinoma. *Hepatology* 66 152–166. 10.1002/hep.29156 28295457

[B34] Zanen-Lim O. G. (1976). Virus-like particles demonstrated by freeze-squeeze technique in acute-phase serum of patients with HBAg-negative hepatitis. *Lancet* 1 18–20. 54519 10.1016/s0140-6736(76)92911-1

[B35] Zhang Z. Sun E. Ou J. H. Liang T. J. (2004). Inhibition of cellular proteasome activities enhances hepadnavirus replication in an HBX-dependent manner. *J. Virol.* 78 4566–4572. 15078938 10.1128/JVI.78.9.4566-4572.2004 PMC387701

