# Supplementary figures and images for: NF-κB-Dependent IFIT3 Induction by HBx Promotes Hepatitis B Virus Replication
Source: Front Microbiol. 2019 Oct 11;10:2382. doi: 10.3389/fmicb.2019.02382 (PMC6797949; doi:10.3389/fmicb.2019.02382)

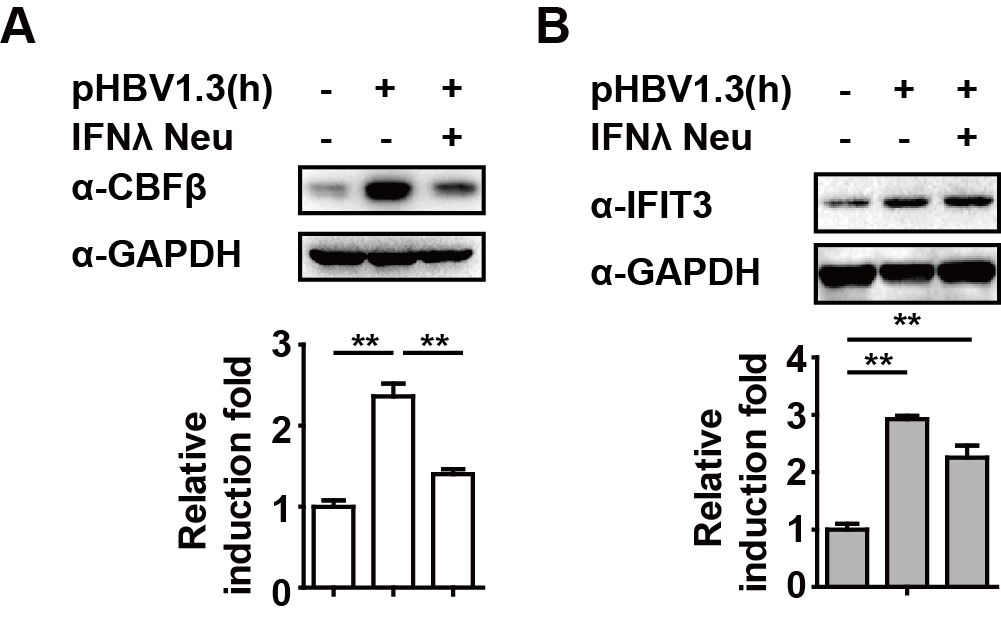

Supplement: FIGURE S1 — (A,B) pHBV1.3 plasmids were transfected into HepG2 cells, and after 42 h these cells were administered with an IFN-γ neutralizing antibody for 6 h, after which qPCR and western blotting were performed to assess the expression of the indicated genes of interest. Data are means ± SD of triplicate experiments, and were compared via Student’s t-test. ∗P < 0.05, ∗∗P < 0.01. [file Image_1.JPEG]

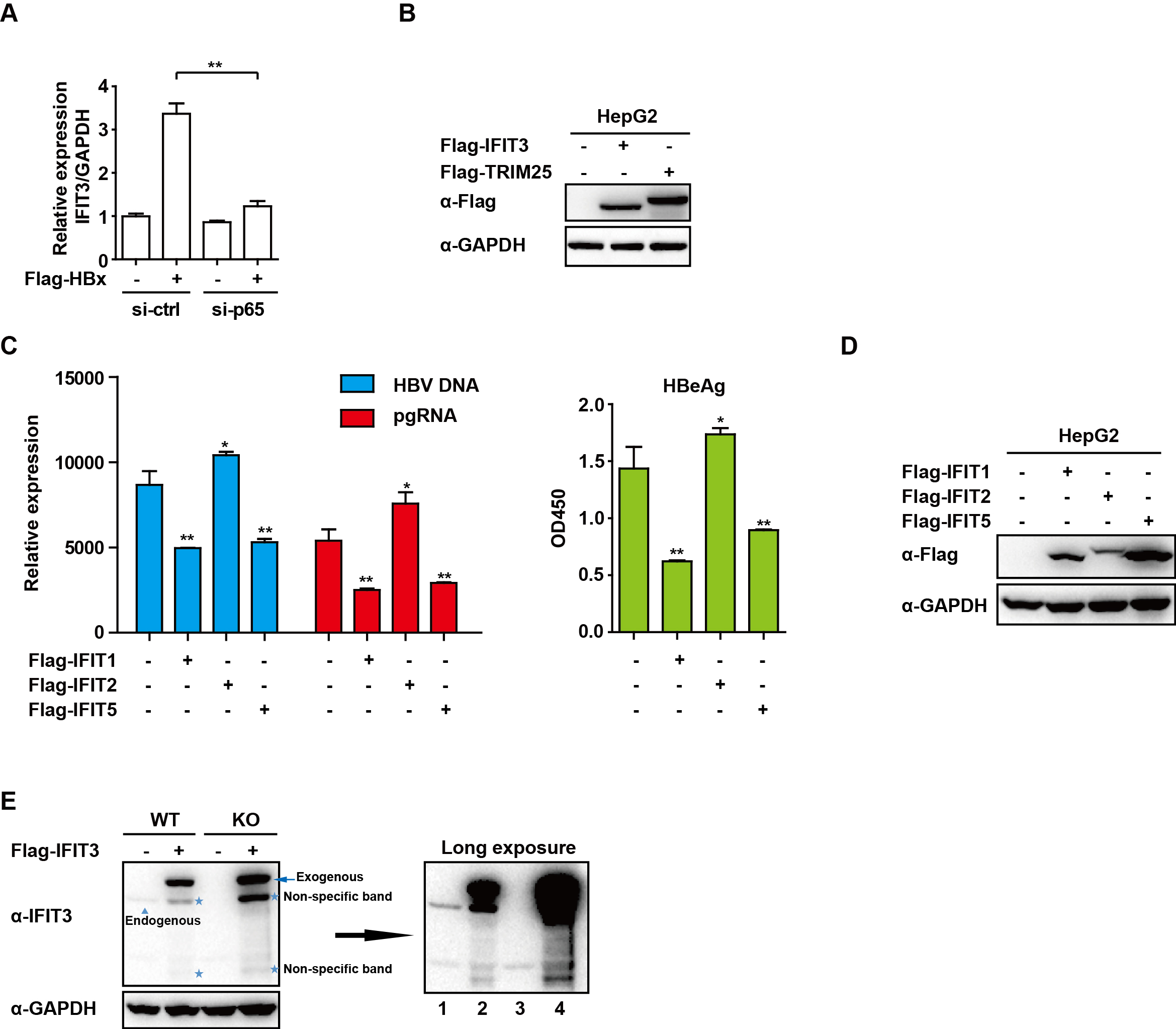

Supplement: FIGURE S2 — (A) IFIT3 mRNA expression was analyzed by Q-PCR. Data are means ± SD of triplicate experiments, and were compared via Student’s t-test. ∗P < 0.05, ∗∗P < 0.01. (B) HepG2 cells were transfected with pHBV1.3 or together with Flag-IFIT1, IFIT2, or IFIT5, and after 72 h cells supernatants were collected. qPCR was used to measure cellular HBV DNA and pgRNA, while supernatant HBeAg levels were measured via ELISA. Data are means ± SD of triplicate experiments, and were compared via Student’s t-test. ∗P < 0.05, ∗∗P < 0.01. (C) Samples from B were immunoblotted with Flag or GAPDH antibodies. (D) Samples from Figure 4A were immunoblotted with Flag or GAPDH antibodies. (E) Samples from Figure 4B were immunoblotted with IFIT3 or GAPDH antibodies. [file Image_2.JPEG]
