# Supplementary material for: NF-κB-Dependent IFIT3 Induction by HBx Promotes Hepatitis B Virus Replication
Source: Front Microbiol. 2019 Oct 11;10:2382. doi: 10.3389/fmicb.2019.02382 (PMC6797949; doi:10.3389/fmicb.2019.02382)
Supplement: TABLE S1 — qPCR Primers. [file Table_1.docx]

**Table S1**

**qPCR Primers**

| **Gene** | **Forward** | **Reverse** |
| --- | --- | --- |
| GAPDH | CGGATTTGGTCGTATTGGG | TCTCGCTCCTGGAAGATGG |
| HBVDNA | GAGTGTGGATTCGCACTCC | GAGGCGAGGGAGTTCTTCT |
| pgRNA | TCTTGCCTTACTTTTGGAAG | AGTTCTTCTTCTAGGGGACC |
| IFIT1 | GCGCTGGGTATGCGATCTC | CAGCCTGCCTTAGGGGAAG |
| IFIT2 | AAGCACCTCAAAGGGCAAAAC | TCGGCCCATGTGATAGTAGAC |
| IFIT3 | AAAAGCCCAACAACCCAGAAT | CGTATTGGTTATCAGGACTCAGC |
| IFIT5 | GGCCAAAATAAAGACGCCCTT | GACCAGGCTTCGTACTTCTTC |

**sgRNAs**

|  | **Forward** | **Reverse** |
| --- | --- | --- |
| IFIT3 sgRNA1 | CACCACACCTAGATGGTAACAACG | AAACCGTTGTTACCATCTAGGTGT |
| IFIT3 sgRNA2 | CACCCGCCTGGGTCTACTATCACT | AAACAGTGATAGTAGACCCAGGCG |

**IFIT3 promoter WT and mutated primers**

|  | **Forward** | **Reverse** |
| --- | --- | --- |
| WT | GGTTGCCAGGCAGGCAACACTTGC | GCACCATGGAATACAATGCATCC |
| Mut1 | GGCAAGGTACCTACCCTGGCCTCT | TACCTTGCCTGAATGCCTACCCTG |
| Mut2 | TGCATACCTACGTCTCTACCCAAA | AGGTATGCAATCAAAACGTCTCTA |
| Mut3 | AACAAATGGTCATGGATAGGAAGA | CCATTTGTTTGTGTCTCATGGATA |

**ChIP primers**

|  | **Forward** | **Reverse** |
| --- | --- | --- |
| P1 | CAGCCTCCTGTTGTCCTG | TGAGAATTTGGCTGTGGG |
| P2 | GCACCTAGATAGGAAGAG | TTGTATCCTGGAACTTTG |
| P3 | ACAGCTAACGCAGGAGGT | TTTGGGTATTTGAGCACTT |
