# Supplementary material for: NF-κB-Dependent IFIT3 Induction by HBx Promotes Hepatitis B Virus Replication
Source: Front Microbiol. 2019 Oct 11;10:2382. doi: 10.3389/fmicb.2019.02382 (PMC6797949; doi:10.3389/fmicb.2019.02382)
Supplement: TABLE S2 — Hepatitis B virus infected patients with IFN treatment. [file Table_2.DOCX]

**Table S2. HBV infected patients with IFN treatment**

| No. | Sex | Age | HBV DNA (copies/mL) | AST (U/L) | ALT (U/L) |
| --- | --- | --- | --- | --- | --- |
| 1 | M | 27 | 170000000 | 188 | 280 |
| 2 | M | 29 | 170000000 | 60 | 106 |
| 3 | F | 31 | 124000000 | 130 | 162 |
| 4 | M | 33 | 170000000 | 78 | 152 |
| 5 | F | 28 | 170000000 | 89 | 145 |
| 6 | M | 29 | 255000000 | 64 | 188 |
| 7 | F | 38 | 170000000 | 88 | 206 |
| 8 | M | 41 | 170000000 | 83 | 154 |
